# Evaluation of potential of *Revstone* ^®^ on gentamicin-induced renal toxicity in Wistar rats: a comparative study of the capsule and syrup forms

**DOI:** 10.3389/fphar.2026.1765648

**Published:** 2026-03-03

**Authors:** Jayshree Shriram Dawane, Priti Dhande, Chandra Dhar Shukla, Aishwarya Dhakne

**Affiliations:** Department of Pharmacology, Bharati Vidyapeeth University Medical College, Pune, India

**Keywords:** acute oral toxicity, nephroprotective, oxidative stress, polyherbal formulation, *Revstone* ^®^

## Abstract

**Introduction:**

*Revstone*®, a polyherbal formulation available in capsule and syrup forms, is designed to treat kidney-related disorders. However, its renoprotective efficacy remains insufficiently validated. In this study, we aimed to evaluate and compare the renoprotective potential of *Revstone*® capsule and syrup formylations in Wistar rats.

**Objective:**

The aim of this study was to assess the acute toxicity profile of *Revstone*® capsule and syrup following Organisation for Economic Co-operation and Development (OECD) guidelines and compare their efficacy in reversing renal damage based on biochemical, histological, and functional markers.

**Methodology:**

Acute oral toxicity testing was conducted according to the OECD 423 guidelines, and rats were observed for 14 days for mortality, behavioural and clinical changes, body weight variations, and gross necropsy findings. For efficacy evaluation, nephrotoxicity was induced by gentamicin (80 mg/kg i.p. for 7 days). Rats were divided into seven groups, namely, the normal control, disease control, *Revstone*® syrup (low and high dose), *Revstone*® capsule (low and high dose), and standard drug control (valsartan 10 mg/kg). Renal function was assessed from serum creatinine, blood urea nitrogen, and uric acid levels. Oxidative stress markers such as malondialdehyde (MDA), superoxide dismutase (SOD), and catalase (CAT) were measured, and histopathological evaluation of kidney tissue was performed. Data were analysed using one-way ANOVA followed by Tukey’s *post hoc* test (*p* < 0.05).

**Results:**

Gentamicin significantly elevated renal markers (*p* < 0.001). *Revstone*® syrup at high dose markedly reduced creatinine (*p* < 0.001), while both formulations improved oxidative stress and renal histology. The syrup formulation was more effective, indicating its potential for future clinical application.

**Conclusion:**

*Revstone*® capsule and syrup demonstrated significant renoprotective activities in gentamicin-induced nephrotoxicity in rats.

## 1 Introduction

Renal disorders, including nephrolithiasis and acute kidney injury (AKI), pose a substantial and escalating burden on global healthcare systems. These conditions not only cause considerable morbidity and mortality but also significantly impact the quality of life and healthcare expenditures.

Nephrolithiasis affects approximately 10%–15% of the global population, and incidences are increasing due to sedentary lifestyles, dietary habits, and climate-related dehydration. It is associated with high recurrence rates, pain, urinary obstruction, and risk of renal impairment if untreated (Tamborino et al., 2024). In many regions, especially in Asia and the Middle East, the prevalence is even higher, reaching up to 20% in certain populations (Kidney stone pathophysiology, evaluation, and management, 2023). Moreover, stone recurrence rates are high, with approximately 50% of patients experiencing a second episode within 5 years, despite treatment (Balawender et al., 2024).

Acute kidney injury, on the other hand, is characterised by a sudden decline in renal function, leading to electrolyte imbalance, metabolic acidosis, fluid overload, and accumulation of nitrogenous wastes, with mortality rates ranging from 10% to over 50%, depending on the severity and comorbid conditions (Martin, 2010). Survivors of AKI are also at increased risk of developing chronic kidney disease (CKD), which further complicates long-term health outcomes.

Despite the availability of pharmacological and surgical interventions, current treatment options for both nephrolithiasis and AKI have several limitations. In the case of kidney stones, alkalising agents, thiazide diuretics, and extracorporeal shock wave lithotripsy (ESWL) are commonly used. However, these approaches often fail to prevent recurrence, and ESWL can cause tissue damage and renal scarring (Loftus et al., 2016). Similarly, in AKI management, the mainstays of therapy include fluid resuscitation, dialysis, and supportive care, which are non-curative and are aimed only at mitigating complications and stabilising the patient. There are no approved nephroprotective drugs that can effectively reverse or prevent AKI (Rumana et al., 2024).

The increasing burden of CKD and AKI has intensified the search for effective nephroprotective therapies, including those derived from traditional medicine (Tamargo et al., 2024). In addition, the shortcomings of current treatment options have fuelled a growing interest in complementary and alternative medicine (CAM) approaches, especially herbal therapies, which have been traditionally used in various cultures to support renal function and treat urinary ailments. Herbal formulations are recognised for their multifaceted mechanisms of action, including antioxidant, anti-inflammatory, antimicrobial, and diuretic effects, which are particularly relevant in mitigating renal injury and stone formation (Kang et al., 2021; Lu et al., 2024). Moreover, herbal drugs tend to have fewer adverse effects, are often more cost-effective, and can be used chronically without significant toxicity (Butterweck and Khan, 2009; Josa et al., 2024).

*Revstone*®, a polyherbal formulation available as a capsule and syrup, is designed to promote renal health and prevent kidney impairment. Its composition includes herbs reputed for their renoprotective properties.

The current study was designed to evaluate both the acute oral toxicity and renoprotective efficacy of *Revstone*® in a preclinical setting using Wistar rats. A well-established experimental model of nephrotoxicity, often induced by chemical agents such as gentamicin, was used to simulate kidney injury. Both capsule and syrup forms of *Revstone*® were tested to assess any formulation-dependent differences in efficacy or safety. Through this comparative approach, the study aims to provide evidence for the nephroprotective potential of *Revstone*®.

## 2 Materials and methods

### 2.1 Animals

Adult male Wistar rats, weighing between 150 and 200 g, were procured from the CCSEA-registered central animal house and housed under standard laboratory conditions (22 °C ± 2 °C, 12 h light/dark cycle) with free access to a standard pellet diet and water *ad libitum*. All experimental procedures were carried out following approval from the Institutional Animal Ethics Committee (IAEC) (IAEC: BVDUMC/751/2025/01/05) and were conducted in accordance with CCSEA guidelines.

### 2.2 Chemicals

Gentamicin 80 mg injection {Troygenta 80 mg/2 mL vial} and valsartan 40 mg tablet {Valtan 40} were both acquired from Bharati Hospital Pharmacy. Enzyme-linked immunosorbent assay (ELISA) and biochemical kits were purchased from BD Biosciences (Qume Drive, San Jose, United States).

### 2.3 Plant material

*Revstone* ^*®*^ syrup- Table 1

**TABLE 1:** *Revstone* ^*®*^ syrup composition. Each 10 mL contains the following ingredients.

Sr. No.	Ingredient	Botanical name	Quantity
1	Betel leaf extract	*Piper betle*	400 mg
2	Cinnamon extract	*Cinnamomum vernum*	50 mg
3	Clove extract	*Syzygium aromaticum*	50 mg
4	Cardamom extract	*Elettaria cardamomum*	50 mg
5	Catechu extract	*Senegalia catechu*	100 mg
6	Black pepper fruit extract	*Piper nigrum*	50 mg
7	Curcumin extract	*Curcuma longa*	50 mg
8	Patharchatta leaf extract	*Bryophyllum pinnatum*	250 mg
9	Cowrie/Cowry shells extract	*Cypraea moneta*	50 mg
10	Punarnava root extract	*Boerhavia diffusa*	250 mg
11	Gokhru fruit extract	*Tribulus terrestris*	250 mg
12	Varun chhal extract	*Crataeva nurvala*	250 mg
13	Hansraj extract	*Adiantum capillus-veneris Linn*	100 mg
14	Key lime extract	*Citrus aurantifolia*	100 gm
15	Calcium oxide extract	—	200 mg

*Revstone* ^*®*^ capsule- Table 2

**TABLE 2:** *Revstone* ^*®*^ capsule composition. Each capsule contains the following ingredients.

Sr. No.	Ingredient	Botanical name	Quantity
1	Pashanbheda	*Saxifraga granulata*	150 mg
2	Shuddha shilajit	*Asphaltum*	100 mg
3	Patharchatta leaf extract	*Bryophyllum pinnatum*	100 mg
4	Patherpori	*Didymocarpus pedicellata*	100 mg
5	Punarnava root extract	*Boerhavia diffusa*	50 mg
6	Ushira	*Vetiveria zizanioides*	50 mg
7	Varuna	*Crataeva nurvala*	50 mg
8	Gokhru fruit extract	*Tribulus terrestris*	50 mg
9	Cowrie shells extract	*Cypraea moneta*	50 mg
10	Nano curcumin	*Curcuma longa*	35 mg
11	Dhaniya	*Coriandrum sativum*	30 mg
12	Daruharidra	*Berberis aristata*	30 mg
13	Shwet chandan	*Santalum album*	30 mg
14	Makoy	*Solanum nigrum*	25 mg
15	Ber patthar	*Aegle marmelos*	25 mg
16	Shatavari	*Asparagus racemosus*	25 mg
17	Khadir	*Acacia catechu*	25 mg
18	Salam mishri	*Polygonatum cirrhifolium*	25 mg
19	Lata karanja	*Caesalpinia crista*	25 mg
20	Guggul	*Boswellia serrata*	25 mg

### 2.4 Acute oral toxicity study

The acute toxicity study was conducted in accordance with OECD Guideline No. 423 (Organisation for Economic Co-operation and Development OECD, 2001). A total of 18 female Wistar rats were randomly divided into groups of three rats per dose. The doses were 500, 1,000, and 2,000 mg/kg, administered orally as a single dose, and the animals were observed individually for 14 days. Syrup formulation was administered orally, while for capsules, the contents were soaked overnight in distilled water, strained the next morning, and homogenised using a stirrer. This was then orally fed to the rats.

#### 2.4.1 Parameters observed

During the study, mortality was continuously monitored for the first 24 h and daily thereafter to detect any treatment-related deaths. Body weights of the animals were recorded on days 0, 7, and 14 to evaluate the potential effects on growth or metabolism. Clinical observations were conducted regularly to identify any signs of toxicity, including changes in skin and fur, eyes, mucous membrane, and alterations in systemic activity and behavioural abnormalities. Retro-orbital blood withdrawal was carried out under ketamine anaesthesia, and after blood collection, the animals were sacrificed by decapitation for histopathological studies of the vital organs. Although no mortality was observed until the end of 14 days, haematological tests (LFT and RFT) were conducted, and a gross necropsy was performed to assess macroscopic pathological changes in major organs, providing insight into any internal effects of the test substance.

### 2.5 Renoprotective efficacy study

#### 2.5.1 Induction of nephrotoxicity

The gentamicin-induced nephrotoxicity model was utilised to evaluate the renoprotective efficacy of *Revstone* ^*®*^ formulations. Nephrotoxicity was induced by administering gentamicin intraperitoneally at a dose of 80 mg/kg for seven consecutive days (Bencheikh et al., 2021).

#### 2.5.2 Experimental design

Rats were randomly divided into seven groups, with six animals in each group, to assess the comparative efficacy of the *Revstone* ^*®*^ formulations. The control group was administered normal saline, while the disease control group was administered gentamicin and treated with normal saline. Doses of *Revstone* ^*®*^ syrup and capsules were extrapolated from the human dose, and the capsules were administered in suspension form prepared with 3% gum acacia. *Revstone* ^*®*^ syrup low-dose and *Revstone* ^*®*^ syrup high-dose groups received gentamicin in combination with low dose (2.7 mL/kg) and high dose (5.4 mL/kg) *Revstone* ^*®*^ syrup, respectively. Similarly, the *Revstone* ^*®*^ capsule low-dose (27 mg/kg) and *Revstone* ^*®*^ capsule high-dose (54 mg/kg) groups were treated with gentamicin along with the respective low and high doses of the *Revstone* ^*®*^ capsule. The standard control group received gentamicin in combination with valsartan (10 mg/kg) as a reference standard.

#### 2.5.3 Drug treatment and blood withdrawal

All animals in groups II to VII were treated with gentamicin for 7 days, and drug treatment was administered from day 2 to day 14. On day 15, blood samples were collected from the retro-orbital plexus under ketamine anaesthesia (80 mg/kg IM). Serum was separated by centrifugation and analysed for key renal function markers, including serum creatinine, blood urea nitrogen (BUN), and uric acid, using standard enzymatic colourimetric methods. In addition, inflammatory markers such as TNF-α and IL-6 were measured (Lee et al., 2015).

#### 2.5.4 Histopathology

Immediately after collecting the blood samples, with animals still under the anaesthetic effects of ketamine, they were sacrificed by decapitation; both kidneys were carefully dissected, rinsed in normal saline, and weighed. One kidney from each animal was processed for histopathological analysis. Tissue sections were stained with haematoxylin and eosin and examined microscopically for pathological changes such as tubular necrosis, glomerular alterations, and inflammatory infiltration. The second kidney was used to assess oxidative stress markers. Renal tissue homogenates were prepared and analysed for malondialdehyde (MDA) levels as an indicator of lipid peroxidation. Antioxidant enzyme activities of superoxide dismutase (SOD) and catalase (CAT) were also measured to evaluate the extent of oxidative damage and the protective antioxidant response within renal tissue.

### 2.6 Statistical analysis

Data were analysed using GraphPad Prism version 10. Statistical analysis was performed using one-way analysis of variance (ANOVA) to determine significant differences among the groups. When a significant F-ratio was obtained, Tukey’s *post hoc* test was applied for multiple comparisons between groups. The results were expressed as the mean ± standard deviation (SD). A *p*-value of less than 0.05 was considered statistically significant throughout the study.

## 3 Results

### 3.1 Results of the acute toxicity study

The study followed OECD guideline 423 using female Wistar rats. A single oral dose of syrup and capsule extract of 500, 1,000, and 2,000 mg/kg was administered, and animals were observed over a 14-day period.

During the acute toxicity study, animals administered both *Revstone* ^*®*^ syrup and capsule formulations at doses of 500 mg/kg, 1,000 mg/kg, and 2,000 mg/kg exhibited no visible signs of toxicity or behavioural abnormalities. All animals showed normal skin, fur, eyes, mucous membranes, and excretory functions. There were no secretions, lacrimation, piloerection, or changes in pupil size. Respiratory patterns and posture remained normal, and gait and response to handling were unaffected. No clonic or tonic movements, excessive grooming, repetitive circling, self-mutilation, or walking backwards were observed throughout the observation period, indicating the absence of any neurological or physiological distress even at the highest tested dose. Food and water consumption were normal throughout the study period, and a normal weight was observed. No mortality or significant clinical signs of distress and no histopathological changes were noted. Relative organ weights remained within the normal limits. Since no change in general parameters and no mortality were observed over the 14-day period in any of the groups, haematological and histopathological studies were conducted only for the syrup group.

#### 3.1.1 Biochemical findings—acute toxicity study

Tables 3, 4.

**TABLE 3:** Effect of *Revstone* ^*®*^ syrup on LFT parameters.

Groups	Total bilirubin (mg/dL)	SGOT (U/L)	SGPT (U/L)	Total protein (g/dL)	ALP (U/L)	Albumin (g/dL)
Dose 1 (500 mg/Kg)	0.7 ± 0.14	242.5 ± 31.8	103.45 ± 1.62	6.85 ± 0.07	121.5 ± 3.25	3.95 ± 0.07
Dose 2 (1,000 mg/Kg)	0.8 ± 0.14	207.25 ± 8.83	97.15 ± 12.37	6.95 ± 0.35	124.55 ± 4.31	3.85 ± 0.07
Dose 3 (2,000 mg/Kg)	0.65 ± 0.07	214.05 ± 38.11	110.3 ± 13.29	6.45 ± 0.49	124.2 ± 2.96	3.9 ± 0.42

Results expressed as the mean ± SD.

There was no significant difference in the LFT parameters of the animals treated with the 500 mg/kg, 1,000 mg/kg, and 2,000 mg/kg doses; *p < 0.05 compared with dose 1 (500 mg).

**TABLE 4:** Effect of *Revstone* ^*®*^ syrup on RFT parameters.

Group	Creatinine (mg/dL)	BUN (mg/dL)	Uric acid (mg/dL)
Dose 1 (500 mg/kg)	0.7 ± 0.28	21.07 ± 0.60	3.26 ± 0.39
Dose 2 (1,000 mg/kg)	0.55 ± 0.21	23.17 ± 4.95	4.39 ± 0.37
Dose 3 (2,000 mg/kg)	0.6 ± 0.14	20.82 ± 1.08	5.06 ± 0.07*

Results expressed as the mean ± SD.

There was no significant difference in the RFT parameters of all the animals treated with the 500 mg/kg and 1,000 mg/kg doses; however, the 2,000 mg/kg dose increased the uric acid level, but it was within the normal limit.

#### 3.1.2 Histopathology—acute toxicity study

**FIGURE 1:**
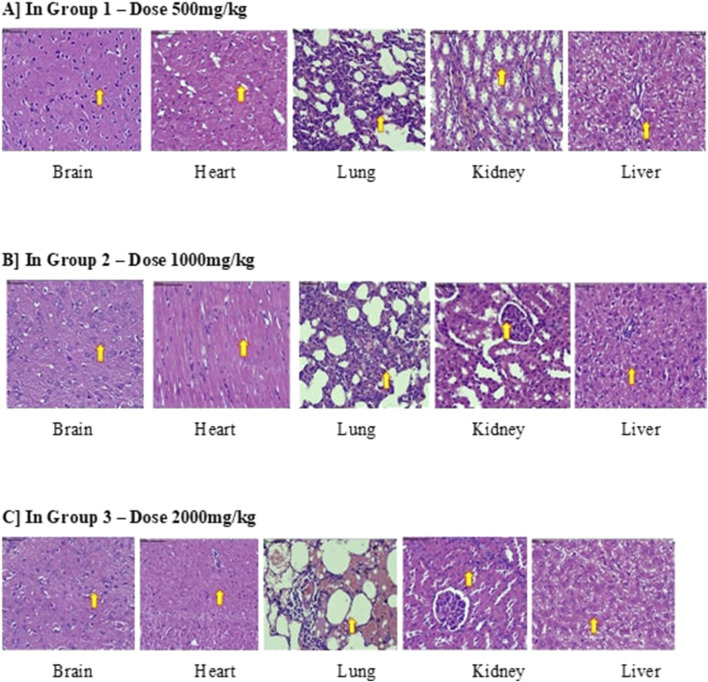
Histopathology of the acute toxicity study of *Revstone* ^*®*^. Microscopic examination of all animals treated with the test item (G1, G2, and G3) did not show any lesion or evidence of toxicity when compared with control animals.

Histopathological examination of the vital organs showed no pathological changes attributable to the test substances at any of the administered dose levels. No clinical signs of toxicity or adverse effects were detected in any group (Figure 1).

### 3.2 Results of the efficacy study

## 4 Discussion

The acute oral toxicity study was conducted to evaluate the safety profile of *Revstone* ^*®*^ syrup and capsule formulations following a single-dose administration in Wistar rats. This assessment is a critical component of preclinical evaluation, intended to identify potential toxic effects and determine the safe dose range before initiating efficacy or chronic toxicity studies. The study was performed in accordance with OECD guideline 423, which utilises a stepwise dosing procedure uses minimal animal numbers, and allows estimation of the LD_50_ range.

Throughout the 14-day observation period, no mortality or treatment-related clinical signs of toxicity were observed in any of the test groups at the administered dose levels of both capsule and syrup formulations. Animals maintained normal behaviour, feeding patterns, and physiological activity. There were no significant changes in body weight gain, indicating the absence of systemic toxicity or metabolic disturbances. This is important because body weight loss of more than 10% in toxicity studies is often considered a sign of adverse effects (OECD, 2001).

Post-mortem gross necropsy revealed no visible pathological changes in vital organs such as the liver, kidneys, heart, lungs, or gastrointestinal tract, indicating that the test formulations did not induce any overt organ-specific toxicity. These findings indicate that *Revstone* ^*®*^ formulations, in both syrup and capsule forms, are well-tolerated and non-toxic at the doses tested.

The absence of acute toxicity indicates that the herbal components of *Revstone* ^*®*^ are safe at the therapeutic and possibly higher doses.

We conducted the acute toxicity study because herbal formulations typically contain multiple phytoconstituents with a wide margin of safety, especially when used in traditional medicine systems, but they also contain minerals and metals in minor quantities. However, it is important to validate their safety through controlled studies to support regulatory compliance and rational clinical use (Khajuria and Jude, 2023).

Overall, the results of the acute toxicity study indicate that *Revstone* ^*®*^ formulations do not produce any immediate toxic effects and are safe for further evaluation in subacute and efficacy studies.

The efficacy study was conducted to evaluate the renoprotective potential of *Revstone* ^*®*^ formulations (syrup and capsule) in a rat model of gentamicin-induced nephrotoxicity. Gentamicin, a commonly used aminoglycoside antibiotic, is well-documented for its nephrotoxic effects, particularly when administered in high doses or over prolonged durations (Bencheikh et al., 2021). The nephrotoxicity primarily results from the accumulation of gentamicin in the renal proximal tubules, where it induces oxidative stress, mitochondrial dysfunction, and apoptosis, ultimately leading to acute tubular necrosis (Klementa et al., 2025).

In this study, repeated administration of gentamicin 80 mg/kg i.p. for 7 days produced significant renal injury, as evidenced by elevated serum creatinine, BUN, and uric acid levels. These biochemical alterations reflect compromised glomerular filtration and tubular reabsorption, which are hallmark features of renal dysfunction (Randjelovic et al., 2017). Additionally, histopathological examination revealed prominent morphological changes, including tubular epithelial degeneration, glomerular shrinkage, and interstitial inflammatory infiltration, which are consistent with previous reports on gentamicin-induced kidney damage (Bencheikh et al., 2021). Oxidative stress is one of the major contributing factors to gentamicin-induced nephrotoxicity. Gentamicin enhances the production of reactive oxygen species, which damage cellular lipids, proteins, and DNA, leading to membrane disruption and cell death. In this context, increased MDA levels serve as a marker of lipid peroxidation, while decreased activities of endogenous antioxidants such as SOD and CAT indicate impaired antioxidant defence mechanisms (Balakumar et al., 2010).

**FIGURE 2:**
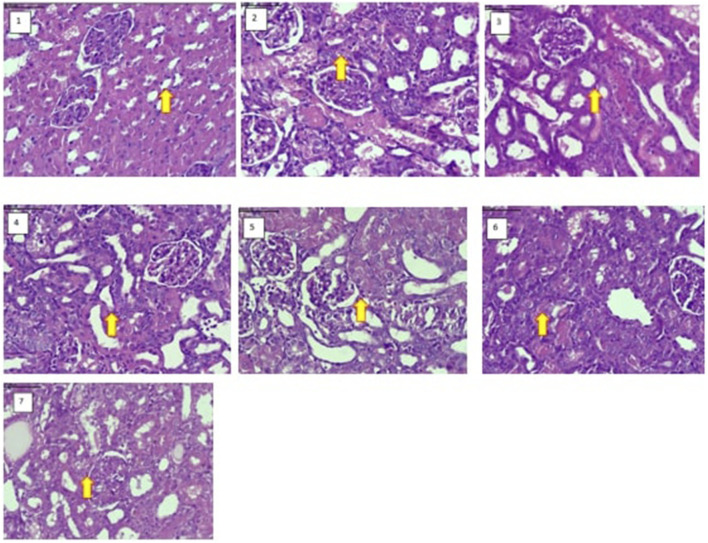
Histopathological findings. Effect of *Revstone* ^*®*^ syrup and capsules on kidney morphological changes under 40 ×, H&E (haematoxylin and eosin) staining. 1) Control, showing normal glomeruli and renal tubules in the cortex and medulla. 2) Disease control, showing vacuolar degeneration and necrosis of renal tubules in the cortex and medulla with multifocal, moderate inflammation. 3) *Revstone* ^*®*^ syrup (low dose), showing basophilic (regenerative) tubules with reduced severity of vacuolar degeneration and necrosis of renal tubules in the cortex and medulla with multifocal, mild inflammation. 4) *Revstone* ^*®*^ syrup (high dose), showing basophilic (regenerative) tubules with reduced severity of vacuolar degeneration and necrosis of renal tubules in the cortex and medulla with multifocal, minimal inflammation. 5) *Revstone* ^*®*^ capsule (low dose), showing basophilic (regenerative) tubules with reduced severity of vacuolar degeneration and necrosis of renal tubules in the cortex and medulla with multifocal inflammation. 6) *Revstone* ^*®*^ capsule (high dose), showing basophilic (regenerative) tubules with reduced severity of vacuolar degeneration and necrosis of renal tubules in the cortex and medulla with multifocal, minimal inflammation. 7) Standard drug (AN), showing basophilic (regenerative) tubules with reduced severity of vacuolar degeneration and necrosis of renal tubules in the cortex and medulla with multifocal, minimal inflammation.

**FIGURE 3:**
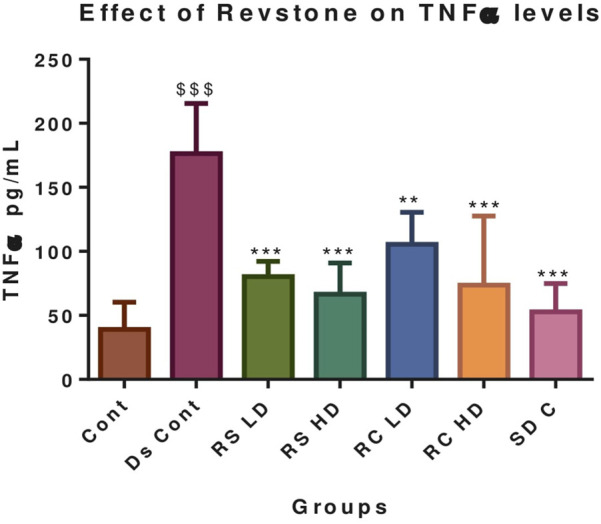
Effect of Revstone^®^ syrup and capsule on serum TNFα. RSLD, *Revstone* ^*®*^ syrup low dose; RSHD, *Revstone* ^*®*^ syrup high dose; RCLD, *Revstone* ^*®*^ capsule low dose; RCHD, *Revstone* ^*®*^ capsule high dose. Values are expressed as the mean ± SD; n = 6; one-way ANOVA followed by Tukey’s test. ^$$$^ *p* < 0.001 compared with the control group, and *p < 0.05, **p < 0.01, and ***p < 0.001 compared with the disease control group. No significant changes in TNF-α levels were observed in the control group. However, the disease control group showed a marked increase in TNF-α levels compared to those in the control group (*p* < 0.001). Treatment with both low and high doses of *Revstone* ^*®*^ syrup significantly reduced TNF-α levels, with the low dose showing significance at *p* < 0.01 and the high dose at *p* < 0.001. Similarly, *Revstone* ^*®*^ capsules at both doses also led to a significant reduction in TNF-α levels (*p* < 0.001). The effect of valsartan was comparable to that of the high-dose *Revstone* ^*®*^ syrup, indicating similar efficacy in lowering TNF-α levels.

**FIGURE 4:**
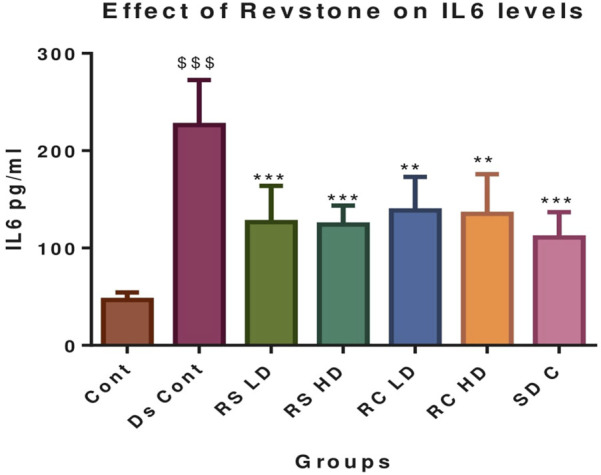
Effect of *Revstone* ^®^ syrup and capsules on serum IL-6. RSLD, *Revstone* ^*®*^ syrup low dose; RSHD, *Revstone* ^*®*^ syrup high dose; RCLD, *Revstone* ^*®*^ capsule low dose; RCHD, *Revstone* ^*®*^ capsule high dose. Values are expressed as the mean ± SD; n = 6; one-way ANOVA followed by Tukey’s test. ^$$$^ *p* < 0.001 compared with the control group, and *p < 0.05, **p < 0.01, and ***p < 0.001 compared with the disease control group. IL-6 levels remained unchanged in the control group. In contrast, the disease control group exhibited a significant elevation in IL-6 levels compared to those in the control (*p* < 0.001). Treatment with *Revstone* ^*®*^ syrup significantly reduced IL-6 levels at both low (*p* < 0.01) and high doses (*p* < 0.001). Similarly, *Revstone* ^*®*^ capsules at both low and high doses also led to a marked reduction in IL-6 levels (*p* < 0.01). The therapeutic effect of valsartan was comparable to that observed with the high-dose *Revstone* ^*®*^ syrup formulations.

Treatment with *Revstone* ^*®*^ formulations resulted in marked improvement in renal function parameters. Both syrup and capsule forms, particularly at higher doses, significantly reduced serum creatinine, BUN, and uric acid levels compared to those in the disease control group (Table 5). These findings indicate the restoration of glomerular filtration and overall renal function. Furthermore, the histopathological evaluation of renal tissue showed a notable reduction in tubular necrosis, cellular degeneration, and inflammation in the treatment groups, indicating structural preservation of the kidney (Figures 2, 3). *Revstone* ^*®*^ reduced the inflammatory markers TNF-α and interleukin-6, demonstrating its anti-inflammatory effects (Figure 4). In addition, its antioxidant potential was evident from its impact on oxidative stress markers (Table 6).

**TABLE 5:** Effect of *Revstone* ^*®*^ syrup and capsule on the renal function parameters and serum calcium.

Group	Creatinine (mg/dL)	BUN (mg/dL)	Uric acid (mg/dL)	Calcium (mg/dL)
Control	0.58 ± 0.11	18.92 ± 2.25	2.46 ± 0.89	8.58 ± 0.31
Ds. control	1.73 ± 0.29^$$$^	24.36 ± 2.46^$$$^	4.10 ± 0.48^$$$^	8.56 ± 0.21
*Revstone*® SLD	1.13 ± 0.43**	17.07 ± 2.93**	2.62 ± 0.55**	8.45 ± 0.24
*Revstone*® SHD	1.01 ± 0.38***	14.30 ± 2.70***	2.33 ± 0.52***	8.71 ± 0.40
*Revstone*® CLD	1.13 ± 0.20**	19.55 ± 1.40**	2.85 ± 0.94*	8.50 ± 0.28
*Revstone*® CHD	1.10 ± 0.30**	18.77 ± 1.18**	2.91 ± 0.50*	8.65 ± 0.18
Valsartan	1.06 ± 0.18**	18.78 ± 1.62**	2.81 ± 0.50**	8.60 ± 0.32

SLD, syrup low dose; SHD, syrup high dose; CLD, capsule low dose; CHD, capsule high dose. Values are expressed as the mean ± SD; n = 6; one-way ANOVA followed by Tukey’s test.

^$$$^ *p* < 0.001 compared with the control group, and *p < 0.05, **p < 0.01, and ***p < 0.001 compared with the disease control group.

No alterations in the measured parameters were noted in the control group. However, the disease control group exhibited a statistically significant (*p* < 0.001) increase in serum creatinine, BUN, and uric acid levels compared to those in the control. Treatment with *Revstone* ^*®*^ syrup at a high dose resulted in a highly significant (*p* < 0.001) reduction in serum creatinine levels, while the low dose also showed a significant decrease (*p* < 0.01) in serum creatinine. The effect observed with the low-dose syrup was comparable to that of both low- and high-dose *Revstone* ^*®*^ capsules and the valsartan-treated group. Among all treatment groups, the high-dose syrup group demonstrated the most pronounced improvement in renal function markers. Serum calcium levels remained unchanged across all the experimental groups.

**TABLE 6:** Effect of *Revstone* ^*®*^ syrup and capsules on oxidative stress parameters in the renal tissue.

Groups	MDA	SOD	Catalase	GSH
Control	6.79 ± 1.47	9.66 ± 0.84	0.113 ± 0.010	11.20 ± 2.57
Ds. control	12.42 ± 0.65^$$$^	4.76 ± 0.57^$$$^	0.057 ± 0.009^$$$^	2.73 ± 1.06^$$$^
*Revstone*® SLD	8.30 ± 1.00***	6.82 ± 1.44**	0.083 ± 0.015*	5.91 ± 1.26**
*Revstone*® SHD	6.31 ± 0.96***	7.23 ± 1.41**	0.087 ± 0.009**	6.67 ± 1.45***
*Revstone*® CLD	8.65 ± 1.03***	6.89 ± 1.24**	0.063 ± 0.021	4.23 ± 0.76**
*Revstone*® CHD	8.15 ± 0.52***	6.84 ± 0.91**	0.070 ± 0.018	6.25 ± 1.84**
Valsartan	7.51 ± 1.23***	7.31 ± 0.50***	0.070 ± 0.018	5.77 ± 1.02**

RSLD, *Revstone* ^*®*^ syrup low dose; RSHD, *Revstone* ^*®*^ syrup high dose; RCLD, *Revstone* ^*®*^ capsule low dose; RCHD, *Revstone* ^*®*^ capsule high dose. Values are expressed as the mean ± SD; n = 6; one-way ANOVA followed by Tukey’s test. ^$$$^ *p* < 0.001 in comparison with the control group, and *p < 0.05, **p < 0.01, and ***p < 0.001 compared with the disease control group.

No significant changes were observed in the levels of MDA, SOD, catalase, and GSH in the control group. However, the disease control group showed a significant (*p* < 0.001) increase in MDA levels, along with a marked decrease in SOD, catalase, and GSH levels (*p* < 0.001), compared to those in the control. Treatment with *Revstone* ^*®*^ syrup at both low and high doses significantly reduced MDA levels (*p* < 0.001). It also enhanced SOD activity in both the doses (*p* < 0.01), increased catalase levels at low dose (*p* < 0.05) and high dose (*p* < 0.01), and elevated GSH levels at low dose (*p* < 0.01) and high dose (*p* < 0.001).

Similarly, *Revstone* ^*®*^ capsules at both doses significantly reduced MDA levels (*p* < 0.001) and increased SOD activity (*p* < 0.01). GSH levels also showed a significant increase at both low and high doses (*p* < 0.01). Although catalase levels increased with capsule treatment, the changes were not statistically significant. The antioxidant effect observed with valsartan was comparable to that of the low-dose *Revstone* ^*®*^ syrup.

*Revstone* ^*®*^ capsule contains nano curcumin, patharchatta, patherpori, punarnava, ushira, varuna, guggul, gokharu, dhaniya, pashanbheda, cowrie shells, shuddha shilajit, daruharidra, shwet chandan, makoy, ber patthar, shatavari, khadir, salam mishri, and lata karanja.

The combination of these botanicals and mineral preparations offers renoprotection through multiple complementary mechanisms. Nano curcumin (*Curcuma longa*), daruharidra (*Berberis aristata*), makoy (*Solanum nigrum*), shatavari (*Asparagus racemosus*), and khadir (*Acacia catechu*) provide potent antioxidant and anti-inflammatory effects, thus reducing oxidative stress, apoptosis, and fibrosis in renal tissues (Ramesh and Radhakrishnan, 2010; Trujillo et al., 2013). Patharchatta (*Bryophyllum pinnatum*) (Pandhare et al., 2021), patherpori (*Didymocarpus pedicellata*), varuna (*Crataeva nurvala*), pashanbheda (*Saxifraga granulata*), and gokharu (*Tribulus terrestris*) exhibit anti-urolithiatic activity, thus preventing crystal deposition, dissolving calculi, and protecting renal tubular epithelium (Singh et al., 2025). Punarnava (*Boerhavia diffusa*) and ushira (*Vetiveria zizanioides*) act as diuretics and anti-inflammatory agents, thus improving glomerular function and reducing proteinuria (Shivananjappa, 2021). Guggul (*Boswellia serrata*) and lata karanja (*Caesalpinia crista*) inhibit pro-inflammatory mediators and protect against renal fibrosis (Zanin et al., 2012; Abdel-Tawab et al., 2011). Mineral-based agents such as shuddha shilajit (asphaltum) and cowrie shells (*Cypraea moneta*) contribute adaptogenic and alkalinising effects, supporting renal resilience (Senturk et al., 2008). Additional herbs such as dhaniya (*Coriandrum sativum*), shwet chandan (*Santalum album*), ber patthar (*Aegle marmelos*), and salam mishri (*Polygonatum cirrhifolium*) provide supportive antioxidant, cytoprotective, and rejuvenating properties (Tsai et al., 2024). Collectively, these agents protect the kidneys by reducing oxidative stress, inflammation, and fibrosis; preventing lithiasis; enhancing diuresis; and restoring renal function, thus demonstrating a holistic renoprotective potential.

Capsule formulation rich in classical Ayurvedic renal herbs such as punarnava, varuna, gokhru, and patharchatta, along with shilajit and antioxidants such as curcumin and daruharidra, emphasises chronic kidney protection through antioxidant, anti-fibrotic, diuretic, and nephroprotective actions, making it suitable for CKD, proteinuria, and toxin-induced nephropathy.

*Revstone* ^*®*^ syrup contains betel leaf, cinnamon, clove, cardamom, catechu, black pepper, curcumin, patharchatta, cowrie shells, punarnava, gokhru, varuna, hansraj, key lime, and calcium oxide.

Many of these herbal and mineral agents exhibit synergistic renoprotective effects through antioxidant, anti-inflammatory, and anti-urolithiatic mechanisms. Betel leaf (*Piper betle*), cinnamon (*Cinnamomum verum*), clove (*Syzygium aromaticum*), cardamom (*Elettaria cardamomum*), and black pepper (*Piper nigrum*) are rich in polyphenols and essential oils that provide antioxidant, antimicrobial, and anti-inflammatory activity, thus protecting renal tissue from oxidative and infectious insults (Fazal et al., 2014; Tjandrawinata et al., 2026). Catechu (*Senegalia catechu*) and curcumin (*Curcuma longa*) exert strong free-radical scavenging and antifibrotic effects, reducing nephrotoxic injury^18^ (Trujillo et al., 2013). Patharchatta (*Bryophyllum pinnatum*), punarnava (*Boerhavia diffusa*), gokhru (*Tribulus terrestris*), and varuna (*Crataeva nurvala*) demonstrate anti-urolithiatic and diuretic activities, thereby preventing stone formation, enhancing urinary excretion, and restoring renal histology (Varalakshmi et al., 1990). Hansraj (*Adiantum capillus-veneris*) and key lime (*Citrus aurantifolia*) contribute additional antioxidant and litholytic effects and are useful in nephrolithiasis (Nazim et al., 2018). Cowrie shells (*Monetaria moneta*) and calcium oxide preparations are traditionally used for urinary alkalinisation and litholysis, thereby reducing crystal aggregation and supporting renal function. Collectively, this formulation offers renal protection by neutralising oxidative stress, reducing inflammation, preventing lithiasis, and improving urinary parameters, thereby preserving kidney function. In the syrup preparation, selected renal herbs and alkalinisers, such as cowrie shells and calcium oxide, are used for urolithiasis prevention, urinary alkalinisation, antimicrobial action, and metabolic support, making it more useful in kidney stone management and early renal protection.

The protective effects observed may be attributed to the phytoconstituents present in *Revstone* ^*®*^, which likely exert antioxidant, anti-inflammatory, and cytoprotective activities. Herbal formulations often contain flavonoids, polyphenols, terpenoids, and other bioactive compounds that can scavenge free radicals, enhance antioxidant enzyme activities, and modulate inflammatory pathways (Vardi et al., 2005; Ghaznavi et al., 2015). The reduction in MDA levels along with a significant elevation in SOD and CAT activities in the *Revstone* ^*®*^-treated groups supports this hypothesis (Table 6). These findings are consistent with earlier studies in which phytotherapeutic agents such as curcumin, silymarin, and glycyrrhizin conferred nephroprotection through similar mechanisms (Cuzzocrea et al., 2000).

The efficacy of *Revstone* ^*®*^ formulations was found to be comparable to that of the standard drug valsartan, an angiotensin II receptor blocker known for its nephroprotective effects. Valsartan not only reduces intra-glomerular pressure but also possesses antioxidant properties that protect renal tissues against oxidative stress and inflammation.

Although this study provides promising evidence of *Revstone* ^*®*^’s nephroprotective action, further studies are needed to isolate and characterise the active phytoconstituents responsible for these effects. Additionally, evaluation in chronic renal injury models and molecular studies targeting oxidative and inflammatory pathways would offer deeper insights into the mechanisms of action and therapeutic applicability in clinical settings.

## 5 Conclusion

The findings of the study indicate that *Revstone* ^*®*^ exhibits significant renoprotective activity, which may be attributed to its anti-inflammatory and antioxidant properties. By reducing pro-inflammatory cytokines such as TNF-α and IL-6 and attenuating oxidative stress, *Revstone* ^*®*^ helps preserve renal function and minimise tissue damage. *Revstone* ^*®*^ syrup preparation was more effective than capsule preparation in this model.

## Data Availability

Authors will provide raw data supporting the article’s conclusions upon reasonable request.
